# Are Congenital Cervical Block Vertebrae a Risk Factor for Adjacent Segment Disease? A Retrospective Cross-Sectional CT and MR Imaging Study

**DOI:** 10.3390/diagnostics12010090

**Published:** 2021-12-31

**Authors:** Cornelius Jung, Patrick Asbach, Stefan M. Niehues

**Affiliations:** 1 Charité—Universitätsmedizin Berlin, Corporate Member of Freie Universität Berlin and Humboldt Universität zu Berlin, Department of Radiology, Hindenburgdamm 30, 12203 Berlin, Germany; corneliusjung@googlemail.com (C.J.); patrick.asbach@charite.de (P.A.); 2 Department of Radiology and Interventional Therapy, Vivantes Klinikum Neukölln, Rudower Str. 48, 12351 Berlin, Germany

**Keywords:** congenital block vertebra, hereditary block vertebra, cervical spine, adjacent segment disease

## Abstract

Adjacent segment disease (ASDI) is a well-described complication of spinal fusion surgery that may ultimately lead to spinal stenosis and repeated surgical intervention. Although congenital block vertebrae also present with degenerative changes in the adjacent segments, this has not yet been systematically investigated. The aim of this study was to assess the presence and degree of ASDI in congenital cervical block vertebrae. Methods: A total of 51 patients with congenital vertebral fusion in one cervical segment were analysed in this IRB-approved retrospective cross-sectional study using available CT/MR imaging. Exclusion criteria were prior spinal surgery and the presence of additional hereditary abnormalities. We assessed the severity of degenerative changes using a sum score. The sum score for adjacent and non-adjacent segments was then divided by the highest possible degeneration score, which resulted in a ratio of severity for adjacent and remaining segments (ranging from 0 to 1). Results: Overall, 35 of 51 patients (68.6%) showed evidence of ASDI, and 34 of 51 patients (66.7%) also showed degenerative changes in the remaining segments. The severity score was significantly higher (*p* = 0.025) in the segments adjacent to the congenital block vertebrae (mean value 0.307) compared to the non-adjacent segments (mean value 0.188). Conclusions: Our results suggest that ASDI is also caused by congenital block vertebrae of the cervical spine.

## 1. Introduction

Congenital block vertebrae are the result of a segmentation disorder in which the chorda dorsalis fails to form the nucleus pulposus, resulting in a rudimentary fibrous intervertebral junction or the complete absence of any disk-like structure [1]. The point of origin is assumed to be the miscoding of the pax-1-gene, which plays a crucial role in the development of the foetal spine between three and six weeks after conception [2]. Congenital block vertebrae have been found in Permian and Triassic temnospondyls (ancient amphibians), suggesting that this spinal abnormality has persisted over hundreds of millions of years [3]. The diagnosis of block vertebra is often incidental on X-ray or cross-sectional imaging (though it is strongly linked to Klippel–Feil syndrome, where visual cues may also be present). Most congenital block vertebrae present in the cervical spine. Morphologically, congenital block vertebrae can be easily differentiated from acquired vertebral fusion. A biconcave shape is seen at the height of fusion (a “wasp-waist”). Rudimentary intervertebral disk material (chorda remnants) may be seen around the zone of fusion, if not a smooth trabecular structure with no signs of scarring. The height of the block vertebra usually equals the combined height of two vertebral bodies plus one intervertebral disk [4], whereas the anteroposterior diameter and the intervertebral foramen is reported to be smaller [5,6]. The vertebral components (body, arch, spinous process) included in the vertebral block may differ, though inclusion of the posterior column shows without doubt that the block is congenital [7,8]. The biomechanical loading of the spine is probably altered in the presence of a congenital block vertebra, which makes the segments adjacent to the block vulnerable to degeneration and therefore potentially clinically relevant. While adjacent segment disease (ASDI) is a well-known complication after spinal fusion surgery [9,10]—the adjacent intervertebral segments show degenerative changes, which may ultimately lead to recurrent spinal stenosis and require repeated surgical intervention [11]—congenital block vertebrae may also present with degenerative changes in the adjacent segments. However, to date, no studies have systematically investigated the degree to which ASDI is associated with congenital block vertebrae. The aim of this study was therefore to assess the presence and degree of ASDI in congenital cervical block vertebrae.

## 2. Materials and Methods

Patient cohort: In this retrospective cross-sectional study, the radiology report database of our hospital was searched for keywords (block vertebra, vertebral fusion, inherited vertebral block, congenital block, spinal fusion), which resulted in pre-screened radiological reports of 2263 patients. The search interval included the years 2000–2015. These radiological reports were subsequently screened for potential congenital block vertebrae, which resulted in 165 patients. The corresponding CTs or MRIs were then reviewed by a musculoskeletal radiologist for the presence or absence of congenital block vertebrae. The inclusion criteria were congenital vertebral fusion and available CT/MR imaging of the cervical spine. Exclusion criteria were prior spinal surgery and the presence of additional hereditary abnormalities (e.g., hemivertebra) or complex hereditary abnormalities of the spine (e.g., butterfly vertebra). We further excluded scans with insufficient image quality and scans that did not include the complete cervical spine. A total of 114 patients were excluded for not meeting the above-stated criteria (screening failure). We finally enrolled 51 patients (26 female, 25 male) with a median age of 43 years (range 12–92 years). The distribution of the 51 block vertebrae per affected segment was as follows: C2/3 (n = 18), C3/4 (n = 5), C4/5 (n = 8), C5/6 (n = 7), C6/7 (n = 7), C7/Th1 (n = 6). Data were analysed after obtaining approval from the institutional ethics committee (EA4/003/15), which waived informed consent given the retrospective study setting. Radiological assessment: All readings were performed by a senior musculoskeletal radiologist and a junior radiologist in consensus using a PACS workstation. The morphology of the congenital block vertebrae was assessed by applying the Brückl classification system [4] (type I–IV; see Supplementary Table S1). In addition, based on the nominal-scale categorisation of the degree of fusion as defined by the Brückl classification system, the degree of fusion per affected component was categorised using the following ordinal-scale sum score (degree-of-fusion score, ranging from 3 to 9, Table 1): (i) *Fusion of intervertebral disk:* normal disk (1 point); hypoplastic disk, without bone contact of corresponding endplates (2 points); hypoplastic disk, with bone contact of corresponding endplates (3 points); complete fusion of vertebral bodies (with or without residual disk) (4 points); (ii) *Fusion of the arches:* both arches not fused (1 point); unilaterally fused arches (2 points); bilaterally fused arches (3 points); (iii) *Fusion of spinous process:* processes not fused (1 point); processes fused (2 points). Subsequently, the presence and degree of degeneration of each cervical spinal segment was assessed as follows utilising CT and/or MRI images.

Based on the following criteria, a sum score for the level of degeneration was calculated (Table 2): (i) *Intervertebral disk degeneration:* loss of height of the intervertebral disk, disk bulging over the dorsal level, evidence of retrospondylophytes (1 point); (ii) *Facet joint degeneration:* joint space narrowing und subchondral sclerosis of the facet joint, hypertrophic yellow ligaments, joint effusion, evidence of osteophytes (1 point); (iii) *Neuroforaminal stenosis:* narrowing through diskal, osseous, or ligamental component (1 point).

In the spinal segment C1/C2 of patients with cervical block vertebrae, evidence of atlantodental degeneration (joint space narrowing, cystic changes of the odontoid process, ligamentous hypertrophy) was evaluated, rather than intervertebral disk degeneration, as well as atlantoaxial degeneration (joint space narrowing, joint effusion, evidence of osteophytes) rather than facet joint degeneration. The sum score was then divided by the highest possible degeneration score (6 for the two adjacent segments and 12 for the four remaining cervical segments) to calculate a ratio subsequently used for comparison of the severity of degeneration. The adjacent spinal segments translate into the adjacent segment ratio and the remaining spinal segments translate into the degeneration ratio. The difference between the two ratios (adjacent segment ratio and degeneration ratio) represents the instability ratio. The instability ratio allowed us to assess whether the presence and degree of degeneration adjacent to the block vertebra exceeded the degeneration of the remaining spinal segments. Statistics: Differences between the adjacent segment ratio and the degeneration ratio were assessed using the Wilcoxon test (significance level *p* < 0.05). A subgroup analysis dividing the patient cohort by age into two groups (age 12–43 years, n = 26 and age 45–92 years, n = 25) was performed using the Mann–Whitney U test. A Spearman’s Rho correlation analysis was used to evaluate the relationship between the three ratios, the patients’ age, and the block-vertebra morphology (Brückl classification and degree-of-fusion score).

## 3. Results

**Figure 1:**
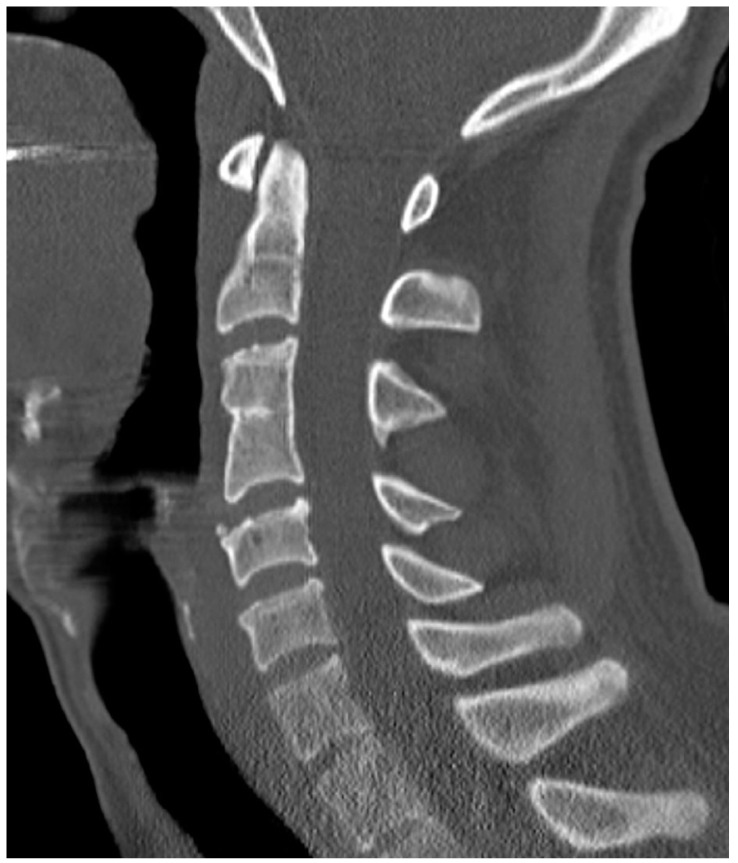
Sagittal CT reconstruction; 28 y/o female; congenital block vertebra C3/4 with degenerative changes in segments C4/5 and C5/6 representing a combination of ASDI and natural degenerative changes.

**Figure 2:**
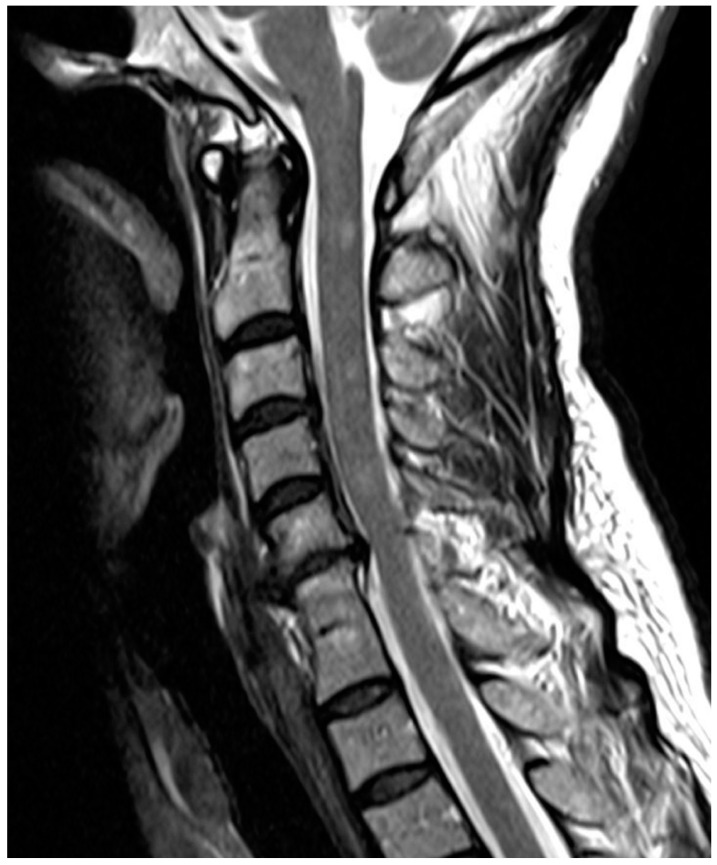
Sagittal T2-weighted fast spin-echo sequence; 53 y/o female; congenital block vertebra C6/7 with degenerative changes in segment C5/6 (disk prolapse) and otherwise normal cervical spine consistent with ASDI. Please also note T2-hyperintense lesions in the cervical spinal cord consistent with demyelination in this multiple sclerosis patient.

**Figure 3:**
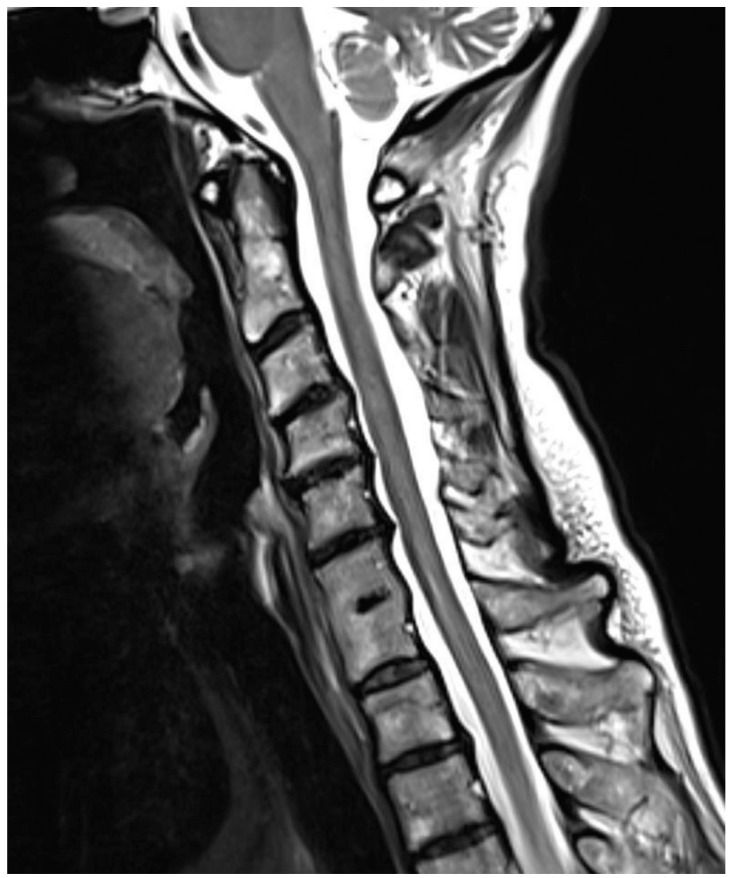
Sagittal T2-weighted fast spin-echo sequence; 59 y/o female; congenital block vertebra C6/7 with degenerative changes in segments C3/4, C4/5, and C5/6 (disk prolapse); however, the segment C7/Th1 is not affected by degeneration, representing a combination of ASDI in one segment and natural degenerative changes. The degeneration score in this patient, who has degeneration in segments C3/4 and C4/5 but not in segments C1/2 and C2/3, is 2 (1 point per segment for loss of height of the intervertebral disk, disk bulging over the dorsal level, and evidence of retrospondylophytes). The respective degeneration ratio is 0.167 (2/12). The degeneration score for the adjacent segments in this patient, who has degeneration in segment C5/6 but not in segment C7/Th1, 1 (loss of height of the intervertebral disk, disk bulging over the dorsal level, and evidence of retrospondylophytes). The adjacent segment ratio is 0.167 (1/6). Consecutively, the instability ratio is 0 (0.167–0.167), which means that the natural degeneration and the degeneration caused by the block vertebra are equal.

According to the Brückl classification, 3 block vertebrae were type II (3/51, 6%), 25 were type III (25/51, 49%), and 23 were type IV (23/51, 45%). The degree-of-fusion score identified 3 block vertebrae that were completely blocked (9 points, 3/51, 6%), 16 block vertebrae that were almost completely blocked (8 points, 16/51, 31%), 2 block vertebrae that were mildly less-blocked (7 points, 2/51, 4%), 4 block vertebrae that were incompletely blocked (6 points, 4/51, 8%), 15 block vertebrae with a mild block (5 points, 15/51, 29%), and 11 vertebrae with a minimal block (up to 4 points, 11/51, 22%). Overall, 35 of 51 patients (68.6%) showed evidence of ASDI (Figure 1, Figure 2 and Figure 3), and 34 of 51 patients (66.7%) also showed degenerative changes in the remaining segments (Figure 1 and Figure 3).

The adjacent segment ratio was significantly higher (*p* < 0.05), measuring 0.307, compared to the degeneration ratio, which measured 0.188 (Table 3).

The instability ratio, which represents the level of degeneration in the segments adjacent to the block minus the level of degeneration of the remaining cervical spine, measured 0.119 (Table 1). The subgroup analysis showed a statistically significant difference regarding the adjacent segment ratio between both age groups (*p* < 0.05); the differences in degeneration ratio and instability ratio were not significantly different between the two age groups but showed a trend towards a difference (*p*-values 0.071 and 0.114, respectively) (Table 1). The Spearman’s Rho correlation analysis showed a significant correlation between the patients’ age and both the adjacent segment ratio (0.544, *p* < 0.01) and the degeneration ratio (0.381, *p* < 0.01); however, no significant correlation was found between the instability ratio (0.213, *p* = 0.113) and patients’ age. Furthermore, no significant correlation was found between the instability ratio and the Brückl score (−0.185, *p* = 0.194) or the degree-of-fusion score (–0.086, *p* = 0.551).

## 4. Discussion

Osteophyte formation, herniation of intervertebral disks, spinal canal stenosis, and luxation in neighboring facet joints are hallmarks of spinal degeneration, which lead to an increase in the biomechanical loading of adjacent spinal segments and consecutively to malalignment and degeneration of neighboring segments [12]. Leisveth et al. [13] investigated 25 patients (with a mean age of 40 years) with cervical block vertebrae in a ten-year follow-up study, radiographically evaluating motion patterns as well as the height of the vertebral body and intervertebral disk in the adjacent motion segments. They describe a significant decrease in the height of the intervertebral disk and vertebral body in the caudally adjacent segment. In a 25-patient radiographic follow-up study (cervical block vertebra C2/C3), Moon et al. [12] found that out of 25 patients, 52% developed spondylosis, whereas only one (4%) patient had spondylosis in the caudally adjacent segments of the block. In a later follow up-study study, Moon et. al. [14] describe 52 patients with cervical block vertebrae, identifying thirteen of them (25%) as having developed ASDI, mostly in the caudally adjacent segments, with only two (4%) involving the immediate caudally adjacent segments, and with some patients reporting ASDI-related symptoms. Further authors describe symptomatic patients with osteophyte formation, spinal canal stenosis, or subluxation in segments adjacent to the block vertebrae, assuming increased loading and biomechanical stress as well as an increase in the motion spectrum (microinstability) in adjacent segments [8,15]. None of these studies, however, quantify their findings or differentiate age-related degeneration from block-vertebra-related degeneration in adjacent segments. We have developed a semi-quantitative score-based approach by calculating severity scores that reflect the presence and degree of degeneration in motion segments adjacent to congenital cervical block vertebrae. The patients in our study had a median age of 43 years, which suggests that patient age played a significant role in the degree of degeneration that we found. Intervertebral disk degeneration has been reported to start as early as 30 years of age, peaking around the age of 40 years [16]. However, our degeneration ratio, which accounts for degenerative changes in all motion segments excluding those adjacent to the block, was significantly lower than the adjacent segment ratio, which represents increased degeneration in the segments adjacent to the block. It can therefore be supposed that the degeneration ratio is directly linked to age and can be counted as the normal age-related degree of degeneration. This is supported by our subgroup analysis, where we divided the cohort into the younger and older individuals, which showed that the degeneration ratio was higher in the older individuals. This contrasts the degeneration bordering the block itself, which is thought to be due to ASDI with an overlap of age-related degeneration. The fact that the ratio for adjacent segments was significantly higher suggests that there is a strong link between congenital block vertebrae and a higher level of spinal degeneration in the adjacent segments (ASDI). The fact that the age subgroup analysis showed no significant difference regarding the instability ratio supports the idea that the congenital block vertebrae inherently affect degenerative changes in the adjacent segments in addition to age-related changes. As mentioned, the specific degree of degeneration was quantified by subtracting the degeneration ratio from the adjacent segment ratio, which gave the instability ratio, representing the surplus degeneration in the segments adjacent to the block vertebra; suspecting the amount of natural degeneration to be relatively equal in the greater area of the block vertebra, we conclude that this surplus degeneration is representative of mainly block-vertebra-related degeneration. A positive instability ratio proves the presence of a surplus of degeneration in the motion segments adjacent to the block vertebra and is therefore a measure of the presence and severity of radiological ASDI. Our conclusions are strengthened by the correlation analysis performed on these results. We found a significant correlation between the patients’ age and both the degeneration ratio and the adjacent segment ratio, meaning that the degeneration that we attributed to a natural cause indeed significantly correlated with the age of the patient, as did the degeneration in segments adjacent to the block. This age dependence was expected, as was the fact that we found no significant correlation between patients’ age and the instability ratio, representing the excess degeneration found in the segments adjacent to the block, as compared to the other segments of the spine. Our statistical analysis therefore also shows that this excess degeneration is not correlated with age and can therefore be assumed to be linked to the block vertebra itself (ASDI). This substantiates our claim that ASDI is found in patients with congenital block vertebra, on the basis of the strong presence of a non-age-related excess of degeneration surrounding the inherited block vertebra. This furthermore supports our idea of this surplus degeneration (ASDI) being mainly due to block-vertebra-related changes in biomechanics. However, our assumption that block vertebra morphology or the degree of fusion has an influence on the degree of degeneration adjacent to the block could not be proven. Although it is believed that a complete fusion of vertebrae would visually—as well as functionally—work as one unit [17], our correlation analysis between block vertebra type and severity was not statistically significant. This does not necessarily imply that there is no connection between the degree of fusion and the degree of degeneration, but it could point to the inability of our model to show a relevant connection between the two. Other experimental models may be more suitable for displaying this type of relationship. It should also be noted that the influence of age on ASDI cannot itself be ruled out and might have caused bias in our study. In fact, some authors assume that patient age plays a vital role in the development of ASDI, claiming that the adjustability of the spine decreases with the rising age of the affected person [12,13,18,19,20]. The degree and the type of affected area (or length of spinal degeneration) are claimed to be directly linked to age, depending on genetic predisposition, nutrition, and individual behavioral patterns [21,22]. The influence of age on ASDI (as represented by our instability ratio, as opposed to our adjacent segment ratio) would be an interesting direction for future research and would build on our findings here. Indeed, we acknowledge that both age-related and block-vertebra-related degeneration are reflected in all of the above-mentioned ratios. We do not believe the age-corrected instability ratio to reflect only ASDI severity, but a combination of both ASDI and natural degeneration; however, here, we expect ASDI to be represented more strongly. It is also important to note that our study was clearly limited by its retrospective design. A prospective follow-up study would be an optimal setting in which to further investigate ASDI, enabling symptom-oriented questioning and functional imaging in a planned time period. However, the rarity of congenital block vertebra makes it very difficult to recruit sufficient numbers of participants for a prospective study design. Moreover, due to the cohort size, we could not evaluate which cervical segments caused which severity of ASDI, since the biomechanical load probably differs between segments. Lastly, we are aware that a calculation of severity scores and correlations does not replace the individual analysis of motion patterns in affected spinal segments. More precise statements could probably be made using functional imaging or biomechanical models in a laboratory setting.

## 5. Conclusions

This study shows that ASDI is likely to develop in congenital block vertebrae of the cervical spine, made evident by the more severe degeneration of the adjacent segments compared to the remaining segments. Therefore, subjects with incidentally diagnosed congenital cervical block vertebrae can be considered a risk group for the development of degeneration, which may be considered when suggesting preventive behavior.

## Figures and Tables

**Table 1:** Degree-of-fusion score (sum score).

Fusion of intervertebral disk	Normal Disk	1 point
	hypoplastic disk, without bone contact of corresponding endplates	2 points
	hypoplastic disk, with bone contact of corresponding endplates	3 points
	complete fusion of vertebral bodies (with or without residual disk)	4 points
Fusion of the arches	both arches not fused	1 point
	unilaterally fused arches	2 points
	bilaterally fused arches	3 points
Fusion of spinous process	processes not fused	1 point
	processes fused	2 points

**Table 2:** Degeneration score (sum score).

Intervertebral Disk Degeneration	Loss of Height of the Intervertebral Disk, Disk Bulging over the Dorsal Level, Evidence of Retrospondylophytes	1 Point
Segment C1/2: Atlantodental degeneration	joint space narrowing, cystic changes of the odontoid process, ligamentous hypertrophy	1 point
Facet joint degeneration	joint space narrowing and subchondral sclerosis of the facet joint, hypertrophic yellow ligaments, joint effusion, evidence of osteophytes	1 point
Segment C1/2: Atlantoaxial degeneration	joint space narrowing, joint effusion, evidence of osteophytes	1 point
Neuroforaminal stenosis	narrowing through diskal, osseous, or ligamental component	1 point

**Table 3:** Quantification of adjacent segment disease in all patients (n = 51) and in the two age groups. Ratios for adjacent segment disease (degeneration in the two segments next to the block vertebra) and ratio for degeneration (degeneration in the remaining cervical segments); the difference between these two ratios represents the instability ratio, quantifying whether the presence and degree of degeneration adjacent to the block vertebra exceeds the degeneration of the remaining spinal segments; SD: standard deviation.

	Mean	SD	Range	Mean (SD) Age Group 12–43 Years	Mean (SD) Age Group 45–92 Years
adjacent segment ratio	0.307	±0.303	0–1	0.192 (±0.274)	0.427 (±0.289)
degeneration ratio	0.188	±0.232	0–1	0.139 (±0.182)	0.240 (±0.268)
instability ratio	0.119	±0.325	0–1	0.054 (±0.284)	0.187 (±0.355)

## Data Availability

The datasets generated and/or analysed during the current study are not publicly available due to the data security regulations of our institution but are available from the corresponding author on reasonable request.

## Supplementary Materials

The following are available online at https://www.mdpi.com/article/10.3390/diagnostics12010090/s1, Table S1: Brückl classification (Brückl, 1979).

## Abbreviations

ASDI	Adjacent segment disease
CT	Computed tomography
IRB	Institutional review board
MRI	Magnetic resonance imaging
PACS	Picture archiving and communication system
