# Supplementary material for: Are Congenital Cervical Block Vertebrae a Risk Factor for Adjacent Segment Disease? A Retrospective Cross-Sectional CT and MR Imaging Study
Source: Diagnostics (Basel). 2021 Dec 31;12(1):90. doi: 10.3390/diagnostics12010090 (PMC8774787; doi:10.3390/diagnostics12010090)
Supplement: Supplementary file 1 [file diagnostics-12-00090-s001.zip › diagnostics-1421645-supplementary.pdf]

Supplementary Table S1:

Brückl classification (Brückl, 1979):

I. Hypoplasia of intervertebral disk. Arches and spinous process remain free (1 Point)

II. Hypoplasia of intervertebral disk. Fusion of arches (Spinous process fused or free) (2 Points)

III. Partial fusion of vertebral bodies (with/or without residual disk) (3 Points)

   a. Arches and spinous process remain free
   b. Arches fused, spinous process remains free
   c. *One arch fused, one arch remains free*

IV. Complete fusion of vertebral bodies (with or without residual disk). Arches and spinous processes fused (4 Points).
